# Leiomyoma of the Foot: A Case Report

**DOI:** 10.7759/cureus.3419

**Published:** 2018-10-05

**Authors:** Katherine Buddemeyer, Haley M McKissack, Chason Farnell, Joseph X Robin, Morad Qarmali, Chandan R Basetty, Perry Washburn, Leonardo V Moraes, Ashish Shah

**Affiliations:** 1 Internal Medicine, University of Alabama at Birmingham, Birmingham, USA; 2 Orthopaedics, University of Alabama at Birmingham, Birmingham, USA; 3 Miscellaneous, University of Alabama at Birmingham, Birmingham, USA; 4 Pathology, University of Alabama at Birmingham, Birmingham, USA; 5 Orthopedics, Instituto De Assistência Médica Ao Servidor Público Estadual, São Paulo, BRA

**Keywords:** heel pain, leiomyoma, foot and ankle, musculoskeletal tumors

## Abstract

Leiomyomas are benign tumors of smooth muscle origin. They are most commonly found in the uterus, but cutaneous leiomyomas may be occasionally present in the extremities and cause pain secondary to mass effect. Few studies have reported leiomyoma of the foot, and leiomyoma of the heel is particularly rare. We present a case of a 41-year-old female who presented to our clinic for a tender nodule on the posterior aspect of her right heel. The tumor was surgically excised and biopsied revealing cutaneous leiomyoma.

## Introduction

Heel pain is a common complaint in the adult population, affecting 17%-24% of people who are 18 years of age and older and often causing significant discomfort and functional impairment [1-2]. The etiologies of heel pain are variable and necessitate generation of a broad differential diagnosis by the physician. Thorough history and physical exam are essential for an appropriate diagnostic workup.

The most common causes of heel pain include plantar fasciitis, Achilles tendonitis, retrocalcaneal bursitis, tarsal tunnel syndrome, and stress fracture [3]. Clinical features such as anatomical location, timing and type of pain, mechanism of injury, and associated physical exam findings can often lead to successful diagnosis [4]. In some cases, history and physical exam are insufficient and imaging is crucial in identifying the causal pathology. Fractures, osteophytes, and congenital bone deformities can be appreciated with simple radiography. Tendinous, ligamentous, and other soft tissue findings are better appreciated with ultrasound and magnetic resonance imaging (MRI). Furthermore, some cases of heel pain warrant further diagnostic techniques such as biopsy and histopathology to identify the underlying cause. An exceedingly rare etiology of heel pain is tumors, which may present as heel pain and/or swelling in the absence of inciting trauma [5].

Leiomyomas are one of the most common benign tumors found throughout the body. Leiomyomas are of smooth muscle origin and are predominantly found in the uterus, affecting up to 80% of women [6]. However, they can possibly be found in any smooth muscle containing organs, including the esophagus and small bowel [7]. Few case reports in the literature describe leiomyomas of the extremities. Although noncancerous, growth of a leiomyoma can elicit a mass effect, compressing local structures and eliciting significant pain. Unlike the majority of pathologies underlying heel pain, leiomyomas require tissue biopsy for diagnosis and surgical excision for definitive treatment [8].

While leiomyoma of the heel is a rare diagnosis, heel pain is one of the most common presenting complaints in clinics across several specialties [4]. Physicians should be aware of the additional steps necessary for appropriate diagnosis and treatment. We present a case of a cutaneous leiomyoma of the posterior aspect of the heel in a young female patient.

## Case presentation

**Figure 1:**
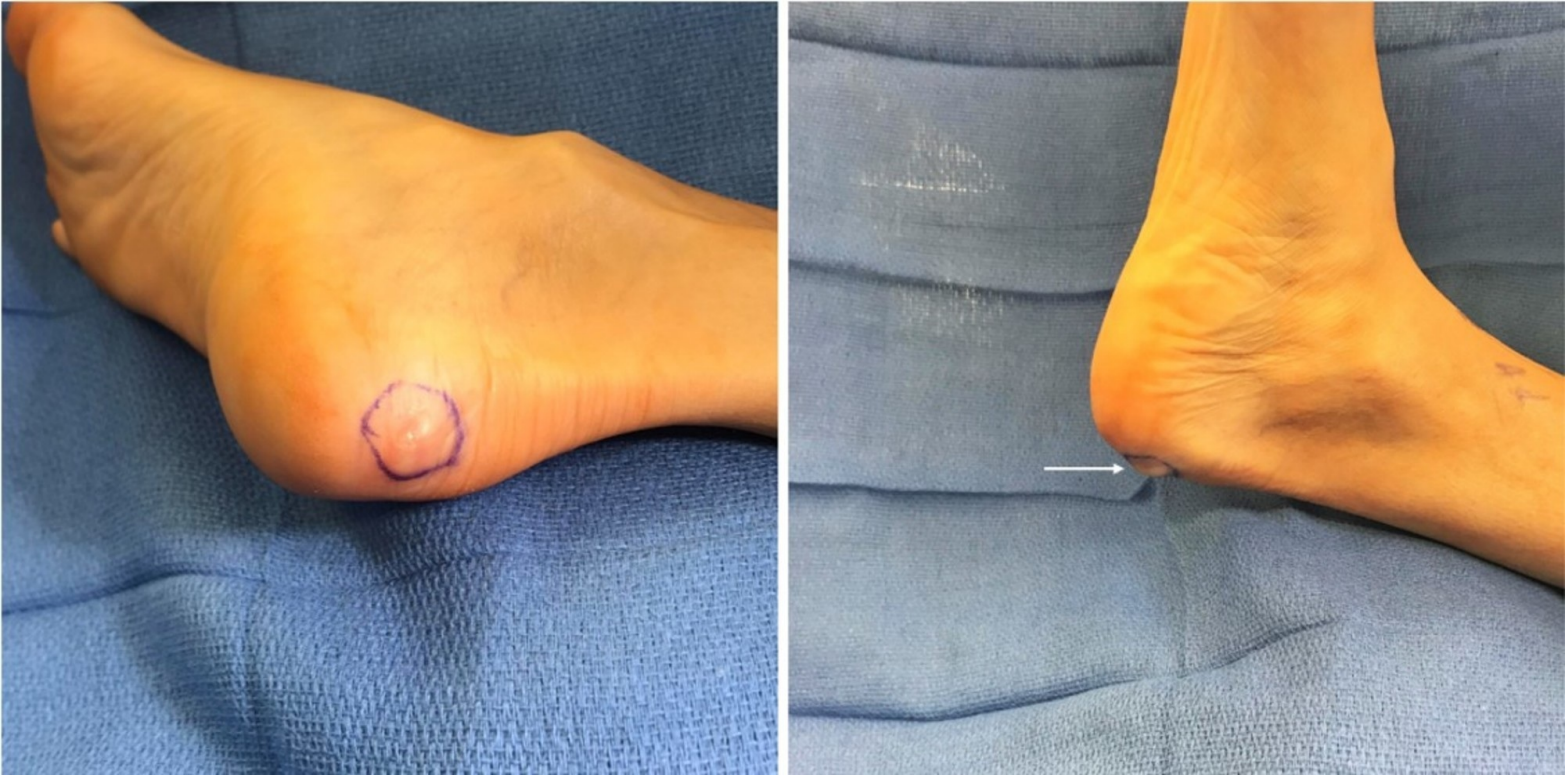
Tumor prior to excision.

A 41-year-old female presented to our clinic as a new patient for excision of a previously diagnosed right heel leiomyoma. Six months prior, she had consulted a dermatologist for a painful area on her right heel that had been present for approximately three to six months. The area was raised, tender, firm, and was increasing in size. Physical exam at that time revealed a 2 cm raised, firm, noncompressible nodule on the posterior right heel, which was tender to palpation without any redness or streaking. X-rays were nonspecific and revealed that the soft tissue shadow was “not connecting to bone.” A punch biopsy taken at a follow-up visit with the dermatologist revealed a leiomyoma of the heel. She continued to experience pain (8/10 in severity) and was seen in our clinic for evaluation six months later. She had no other areas of bleeding, itching, or pigment changes of the skin. Physical exam revealed a palpable 2 cm x 1 cm nodule on the posterior aspect of the right heel which was freely mobile and tender to palpation (Figure 1). Pulses and sensation were normal throughout in both lower extremities and no other gross abnormalities were noted.

The patient did not have a history of frequent running, gout, diabetes, constitutional symptoms, or weight loss. Due to the unusual location of this leiomyoma, Reed’s syndrome was considered in the differential diagnosis, and the patient was referred for a gynecological oncology appointment. However, because she had no personal or family history of other similar soft tissue masses, uterine fibroids, renal cell carcinoma, or skin cancer, there was a low index of suspicion. Her past medical history was nonspecific for any condition predisposing to cancer.

**Figure 2:**
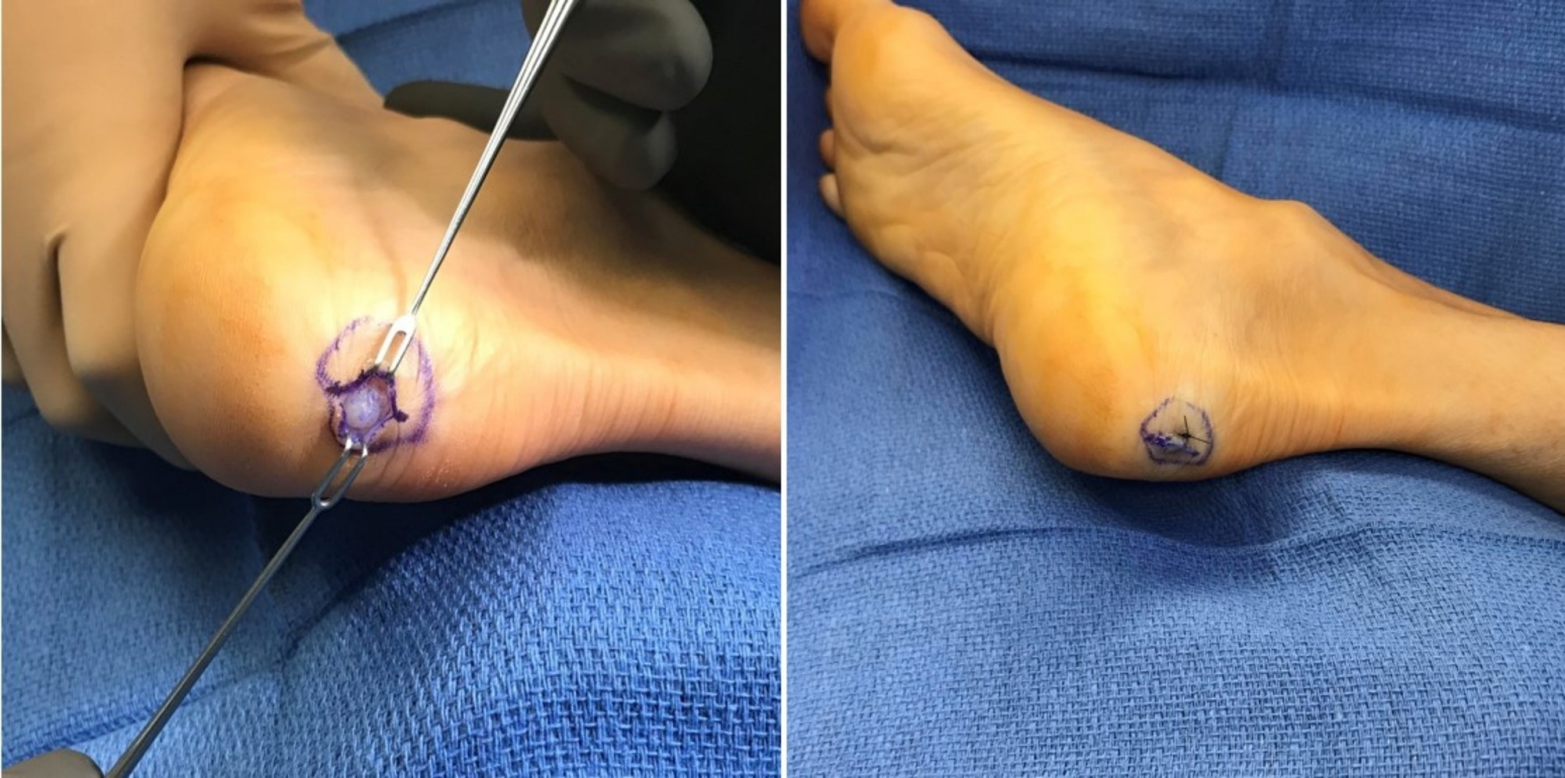
Excision of tumor. Exposed tumor prior to resection (left) and closed wound after resection (right).

**Figure 3:**
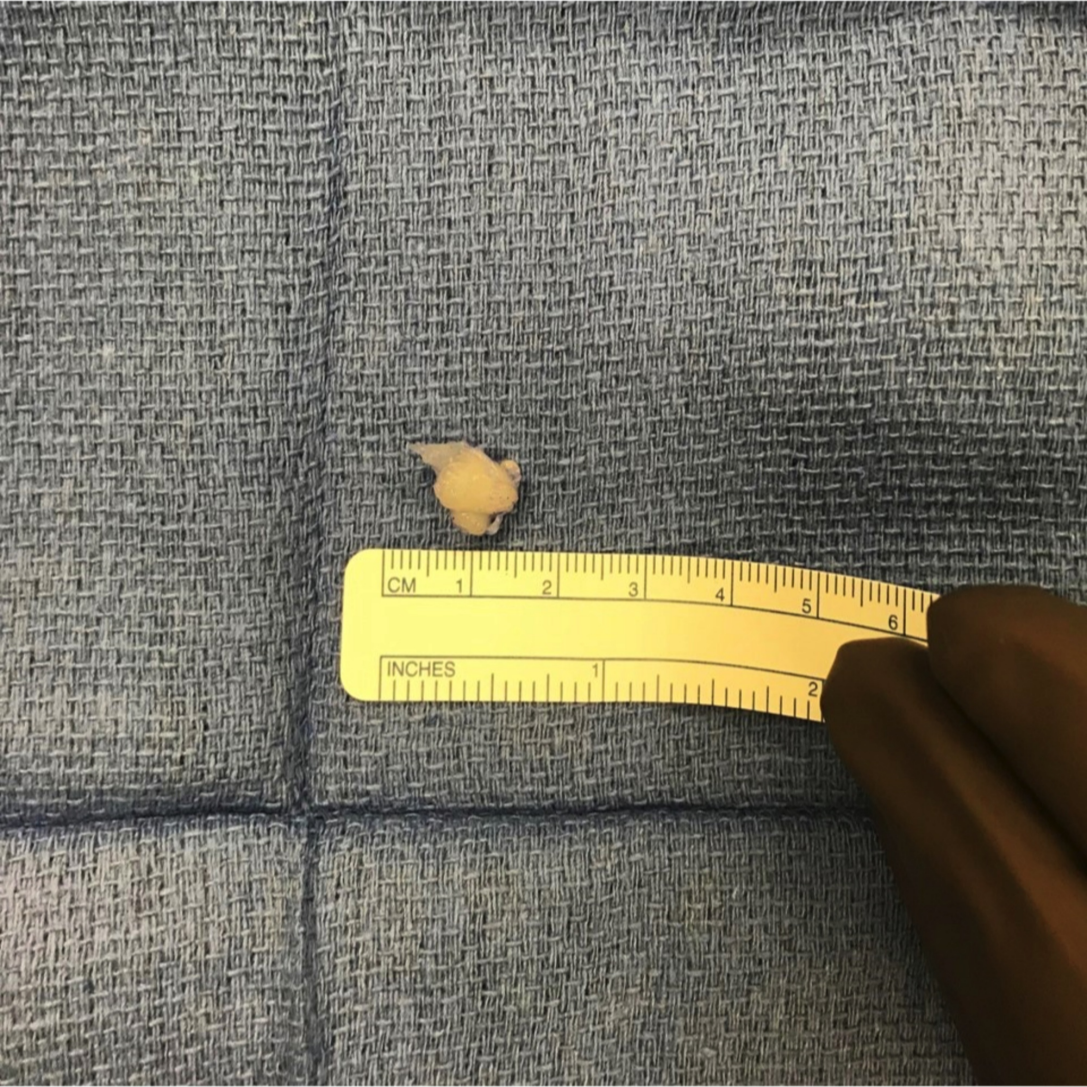
Excised tumor.

**Figure 4:**
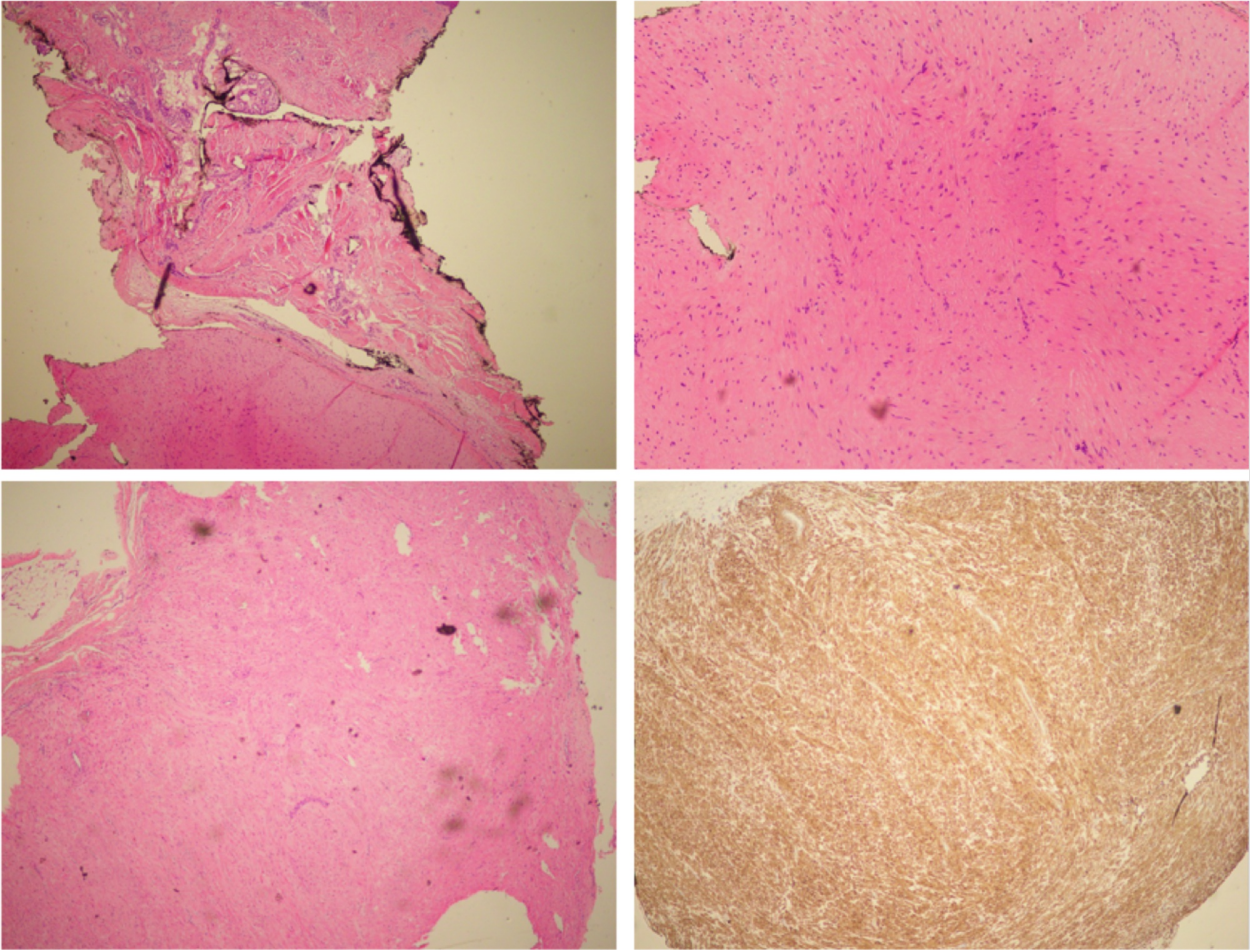
Histopathological sections of excised tumor confirming diagnosis of leiomyoma. Leiomyomas are benign smooth muscle tumors that are common and rarely transform into its malignant counterpart, leiomyosarcoma. The tumor can arise at any organ. The most common location is the uterus. Other common locations are the skin and the gastrointestinal tract. Histopathology sections of the tumor at both locations exhibit similar histology. The tumor consists of bundles and fascicles of well-differentiated smooth muscle cells with eosinophilic cytoplasm, cigar-shaped nuclei, and abundant extracellular matrix. Immunohistochemical staining for caldesmon, a smooth muscle specific antibody, supports the diagnoses of leiomyomas.

She was scheduled for surgery two weeks later. The tumor was excised (Figures 2-3) and sent for pathology confirming cutaneous leiomyoma (Figure 4). Follow-up visit at two weeks status post-excision showed a well-healing incision with no signs of infection, erythema, or discharge, and no signs of any nodule remaining.

## Discussion

Leiomyomas are benign smooth muscle tumors rarely reported outside the uterus and gastrointestinal tract. Cutaneous leiomyomas can be divided into three subcategories based on the tissue of origin: angioleiomyomas, piloleiomyomas, and genital leiomyomas. Angioleiomyomas and piloleiomyomas are the most common subtypes of cutaneous leiomyoma, both of which may occur over the extremities. Due to the nonspecific clinical nature of cutaneous leiomyomas, the diagnosis is primarily based upon histological features [9].

Angioleiomyomas (synonymous with angiomyoma and vascular leiomyoma) arise from the tunica media of venous vasculature and are the clinical sub-type of cutaneous leiomyoma most likely to present in the extremities. Typically, they develop as a small (<2 cm) solitary lesion, and more than half of cases present with pain or tenderness. Two-thirds occur between the fourth and sixth decades of life, and there is a 2:1 predilection for females [10]. In contrast to angioleiomyomas, piloleiomyomas (synonymous with pilar leiomyoma) arise from the arrector pili muscle of the hair follicle. They commonly present as multiple red-brown, firm, rounded nodules on the extensor surfaces of the extremities, and are also frequently painful. Although solitary piloleiomyomas are rare, they may develop on the lower extremities. Unlike angioleiomyomas, piloleiomyomas usually arise in adolescence to early adulthood and affect men and women equally [9].

Cutaneous leiomyomas raise suspicion for an underlying etiology of hereditary leiomyomatosis and renal cell cancer (HLRCC), also known as Reed’s syndrome. HLRCC is caused by an autosomal-dominant inactivating mutation of the tumor supressor fumarate hydratase [11]. The result is the formation of cutaneous leiomyomas and renal cell carcinomas, as well as uterine leiomyomas in women. Piloleiomyomas , especially when numerous, have the strongest association with HLRCC; however, all subtypes of cutaneous leiomyomas may be present with HLRCC. Identification of cutaneous leiomyomas should raise clinical suspicion for HLRCC and genetic testing should be performed if the patient meets the HLRCC diagnostic criteria [12].

Our case does capture the most frequently observed presentation of cutaneous leiomyoma, arising as a small, solitary, tender nodule on the heel of a 41-year-old female. However, the differential diagnosis of heel pain includes a relatively long list of benign and malignant etiologies. Mechanical causes, including plantar fasciitis, nerve entrapment, calcaneal spur, and stress fracture, are much more often responsible for heel pain. Overall, tumor is a rare cause [13].

Only an estimated 5% of all malignant soft tissue lesions and 8% of all benign soft tissue lesions occur at the foot or ankle [13] and there is a significant variety in the types of soft tissue tumors that affect the foot. Origins include adipose, smooth and skeletal muscle, fibrous tissue, vasculature, nerves, and primitive mesenchyme. Various locations on the foot tend to develop different types of tumors with the heel being a particularly infrequent site of tumor involvement. In a rare instance of the tumor at the site of the heel, the most common malignant neoplasms include clear-cell sarcoma, Kaposi’s sarcoma, and malignant fibrous histiocytoma. The most common benign neoplasms of the heel include giant-cell tumor, lipoma, and leiomyoma [13]. Nevertheless, given the overall low incidence of soft tissue foot tumors, especially on the heel, even these most common neoplasms are infrequently diagnosed. 

## Conclusions

Despite the overall low incidence of heel tumors, leiomyoma should be considered in the differential for heel pain in the absence of apparent mechanical etiology. As demonstrated in our case, identification of leiomyoma offers a positive prognosis. Surgical excision can be curative for solitary lesions and provides complete symptom relief.
